# Geographic and Socioeconomic Disparity in Child Undernutrition across 514 Districts in Indonesia

**DOI:** 10.3390/nu14040843

**Published:** 2022-02-17

**Authors:** Dumilah Ayuningtyas, Dwi Hapsari, Rika Rachmalina, Vilda Amir, Riani Rachmawati, Dian Kusuma

**Affiliations:** 1 Health Policy and Administration Department, Faculty of Public Health, Universitas Indonesia, Depok 16424, Indonesia; dumilah@gmail.com (D.A.); vildaamir@gmail.com (V.A.); 2 National Institute of Health Research and Development, Ministry of Health, Jakarta 10560, Indonesia; dhapsari2001@yahoo.com (D.H.); rika.rachmalina@gmail.com (R.R.); 3 Faculty of Economics and Business, Universitas Indonesia, Depok 16424, Indonesia; riani.rachmawati@ui.ac.id; 4 Centre for Health Economics & Policy Innovation, Imperial College Business School, London SW7 2AZ, UK

**Keywords:** undernutrition, malnutrition, wasting, stunting, Indonesia, geospatial

## Abstract

Background: Globally, in 2020, 45 million children were estimated to be wasted, and 149 million children under five years of age were estimated to be stunted. Undernutrition makes children in particular much more vulnerable to disease and death. Our study aims to examine geographic and socioeconomic disparities in child undernutrition across 514 districts in Indonesia. Methods: Employing both geospatial and quantitative analyses (descriptive statistics and Ordinary Least Squares regressions), we analyzed the disparities in the prevalence of underweight, severe underweight, wasting, severe wasting, stunting, and severe stunting among districts. Child undernutrition data were from Indonesia Basic Health Survey (Riskesdas) 2018, which included a sample of 93,620 children under five years. Socioeconomic data were from the World Bank. Results: We found a relatively large geographic and socioeconomic disparity in child undernutrition in Indonesia. By region, districts in the Papua region (including Maluku and Nusa Tenggara) had a significantly higher prevalence of underweight and wasting than those in the Java region (including Bali). Districts in Papua had 44%, 121%, 38%, and 57% higher prevalence of underweight, severe underweight, wasting, and severe wasting, respectively. Similarly, the poorest districts had a significantly higher prevalence of underweight, wasting, and stunting than the wealthiest districts. The poorest districts had 30%, 83%, 16%, 21%, and 74% higher prevalence of underweight, severe underweight, wasting, stunting, and severe stunting, respectively. These results were similar among rural districts. Conclusion: There is a significant disparity in child undernutrition across districts in Indonesia. The government needs to prioritize the reduction of child undernutrition, especially in rural areas, districts outside of Java and Bali, and the poorest and least educated areas.

## 1. Background

Globally, in 2020, 45 million children were estimated to be wasted (too thin for their height), and 149 million children under five years of age were estimated to be stunted (too short for their age). Undernutrition makes children in particular much more vulnerable to disease and death. Around 5% of deaths among children under five are linked to undernutrition, and these mostly occur in low- and middle-income countries (LMICs) [1]. Moreover, the COVID-19 pandemic is expected to exacerbate child undernutrition and child mortality, especially in LMICs. A modeling study estimated that in 2022, COVID-19-related disruptions could result in an additional 9.3 million wasted children, 2.6 million stunted children, and 168,000 additional child deaths [2].

In Indonesia, a lower-middle-income country, the burden of child undernutrition is similarly high. The latest data from Indonesia Basic Health Survey (RISKESDAS), a nationally representative sample, showed that 7.2% and 19.7% of children under five were wasted and stunted, respectively [3]. With an estimated 24 million children under five in the country, over 1.7 million and 7.4 million children were wasted and stunted. While data showed that under-five mortality rates decreased from 52.2 per 1000 live births in 2000 to 23.9 in 2019, it was still higher than in many neighboring countries such as Malaysia (8.6 per 1000 live births), Thailand (9.0), and Vietnam (19.9) in 2019 [4].

The linkage between socioeconomic environments and child undernutrition has been well-studied. A study using data from 47 LMICs in Asia, Africa, and Latin America showed pronounced within-country socioeconomic inequalities in stunting and wasting among children under five years old [5]. The study showed that in Pakistan, for instance, the prevalence of stunting was 58.2% and 30.6% among the poorest and wealthiest quintiles, respectively. In Guatemala, the prevalence of stunting was 68.5% and 25.5% among the poorest and wealthiest quintiles, respectively [5]. Another study analyzed data from 35 sub-Saharan African countries and found that underweight, wasting, and stunting were significantly higher among children from lower-income households, mothers with lower education, and rural areas [6]. Furthermore, previous studies have also shown some evidence on geographic disparity in child undernutrition. A study in India analyzed data across 640 districts in India and found that the prevalence of stunting was higher among poorer populations in northern India compared to eastern and southern parts of India [7]. Additionally, a study in Argentina found regional disparities with higher prevalence values in the north and northeast regions [8]. A better understanding of these disparities provides a precision public health tool to target public policies to those populations with the greatest need in order to reduce health disparities [8].

Narrowing the geographic and socioeconomic disparity in child undernutrition is crucial to achieving the Sustainable Development Goals of reducing wasting and stunting. However, previous studies on the geographic and socioeconomic disparity in child undernutrition from LMICs are limited in at least three ways. First, the majority of studies used individual-level data (including Demographic Health Surveys and Family Health Surveys) to examine the socioeconomic disparity or inequality, including studies from Asia (including India, Bangladesh, Nepal, Cambodia, and Indonesia), Africa (including Nigeria, Chad, and Sierra Leone), and Latin America (including Brazil, Guatemala, and Argentina) [6,7,8,9,10,11,12,13,14,15,16,17,18,19]. While that evidence is invaluable, analyses using locality-level data (including districts) are also needed to inform policies. For instance, district-level studies for Indonesia (and other countries with similar settings) are crucial because of the decentralization policy, which transfers health sector planning to district heads. Second, while there are plenty of studies on socioeconomic disparity, those on geospatial patterns are lacking [7,8]. Thus, our study aims to address this evidence gap by examining the geographic and socioeconomic disparity in child undernutrition (underweight, wasting, and stunting) across 514 districts in Indonesia, using data from a nationally representative health survey.

## 2. Methods

### 2.1. Study Design and Sample

We conducted a cross-sectional study on the geographic and socioeconomic disparity in child undernutrition (under five years old) among districts in Indonesia. Geospatial analyses were performed to assess geographic variations of child undernutrition. Additionally, quantitative regression analyses were conducted to examine the associations between socioeconomic indicators and child undernutrition. The child undernutrition data were from RISKESDAS 2018, a nationally representative health survey by the Ministry of Health, aggregated at the district level (514 districts within 34 provinces).

The target sample of RISKESDAS was 300,000 households from 30,000 census blocks from the National Socioeconomic Survey with two-stage sampling. First, the survey team selected 180,000 census blocks using probability proportional to size from 720,000 census blocks listed in the population census 2010. Additionally, then the team selected 30,000 census blocks in each urban and rural using probability proportional to size. Second, the team systematically chose ten households using implicit stratification of the education level of household heads (to maintain the variation among households). The team interviewed each household member and examined participants meeting the inclusion criteria. The interview response rate in RISKESDAS was relatively high at 95% of target households nationally (ranging from 85% in Papua province to 99% in Bangka Belitung province). The sample included 93,620 children under five years, 818,507 individuals aged 10+ years, and 713,783 individuals aged 15+ years [3].

### 2.2. Independent Variables

**Figure 1:**
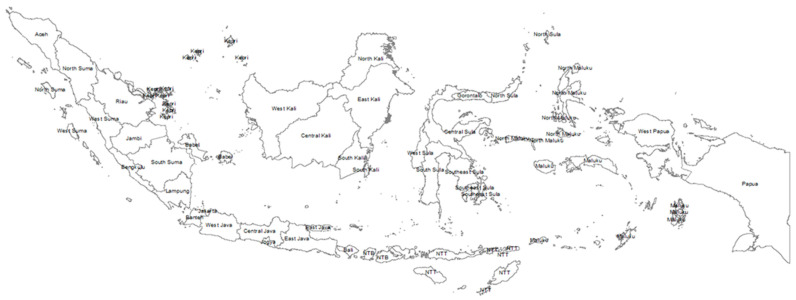
Map of Indonesia by province. Note: Suma = Sumatera, Kepri = Riau Islands, Sula = Sulawesi, Kali = Kalimantan, NTB = West Nusa Tenggara, NTT = East Nusa Tenggara. We divided the provinces into five regions including Sumatera, Java/Bali, Kalimantan, Sulawesi, and Papua/Maluku/Nusa Tenggara. The latter region is the least developed. The Indonesian Information and Geospatial Agency provided the shapefile; the authors created the map in ArcMap 10.

The socioeconomic indicators were obtained from the World Bank database. The districts and provinces were grouped into five regions: Sumatera, Java (including Bali), Kalimantan, Sulawesi, and Papua (including Nusa Tenggara and Maluku) see Figure 1. The Java region is the most developed economically, and the Papua region is the least developed [20,21,22]. By income, the district-level poverty rates were used and grouped into five quintiles, with the highest poverty rate as the first quintile (i.e., poorest) and the lowest poverty rate as the last quintile (i.e., wealthiest). By education, net enrollment ratios of senior secondary were used and grouped into five quintiles, with the first quintile as the least and the last quintile as the most educated. Socioeconomic data were used for all 514 districts, but child undernutrition data were used for 513 districts. Yalimo regency had missing values because of lacking children under five in the sample. The analyses were conducted using overall districts and urban/rural areas (cities as urban and regencies as rural) [20,21,22].

### 2.3. Dependent Variables

Six indicators of child undernutrition were used as dependent variables: underweight, severe underweight, wasting, severe wasting, stunting, and severe stunting. The cut-off values were compared with the median of the WHO child growth standards for each indicator. Underweight and severe underweight were defined as a weight for age z-score (WAZ) less than −2 standard deviations and −3 standard deviations, respectively. Wasting and severe wasting were defined as height for age z-score (HAZ) of less than −2 standard deviations and −3 standard deviations, respectively. Additionally, stunting and severe stunting were defined as a weight for height z-score (WHZ) of less than −2 standard deviations and −3 standard deviations, respectively [3].

Wasting usually indicates recent and severe weight loss because a child has not had enough food to eat or has had an infectious disease, such as diarrhea, that has caused them to lose weight. Stunting is due to chronic or recurrent undernutrition, usually associated with poor socioeconomic conditions, poor maternal health and nutrition, frequent illness, and inappropriate infant and young child feeding and care in early life. Stunting holds children back from reaching their physical and cognitive potential. Underweight children have low weight for their age and may be stunted, wasted, or both [1].

### 2.4. Data Analysis

Geospatial analyses were conducted by dividing the undernutrition prevalence among 34 provinces and 514 districts into five quintiles in ArcMap 10. Dark red and red color systems were used to show the provinces and districts in the fifth quintile (highest prevalence of undernutrition) and the fourth quintile (higher prevalence), respectively. Dark blue and blue color systems were used to show the provinces and districts in the first quintile (lowest prevalence) and the second quintile (lower prevalence), respectively. This was done for each outcome variable.

Moreover, quantitative analyses were performed using descriptive statistics and bivariate analysis. Descriptive statistics included the prevalence of undernutrition by province and district as well as district characteristics by socioeconomic variables. Bivariate Ordinary Least Square (OLS) regressions in STATA 15 were performed to show associations between geographic (i.e., urban/rural and region) and socioeconomic (i.e., income and education) disparity for each undernutrition indicator: underweight, severe underweight, wasting, severe wasting, stunting, and severe stunting. The absolute and relative differences were calculated between geographic and socioeconomic variations. By region, the absolute and relative differences were between the Papua region (least developed) and the Java region (most developed). By income, the absolute and relative differences were between quintile 1 (poorest) and quintile 5 (wealthiest). By education, the absolute and relative differences were between quintile 1 (least educated) quintile 5 (most educated). All statistical significance was at the 5% level.

## 3. Results

The results were presented at the provincial and district levels. While the evidence on the disparity in child undernutrition at the provincial level is relevant for national development planning, the small number of provinces (i.e., 34 provinces) limits the observations for quantitative analysis. On the other hand, the large number of districts (i.e., 514 cities and regencies) is sufficient for further quantitative analysis. In terms of policy, district-level studies for Indonesia (and other countries with similar settings) are crucial because of the decentralization policy, which transfers health sector planning to district heads. Thus, district health offices are accountable to local district heads, not the national Ministry of Health.

### 3.1. Provincial Level Analysis

**Figure 2:**
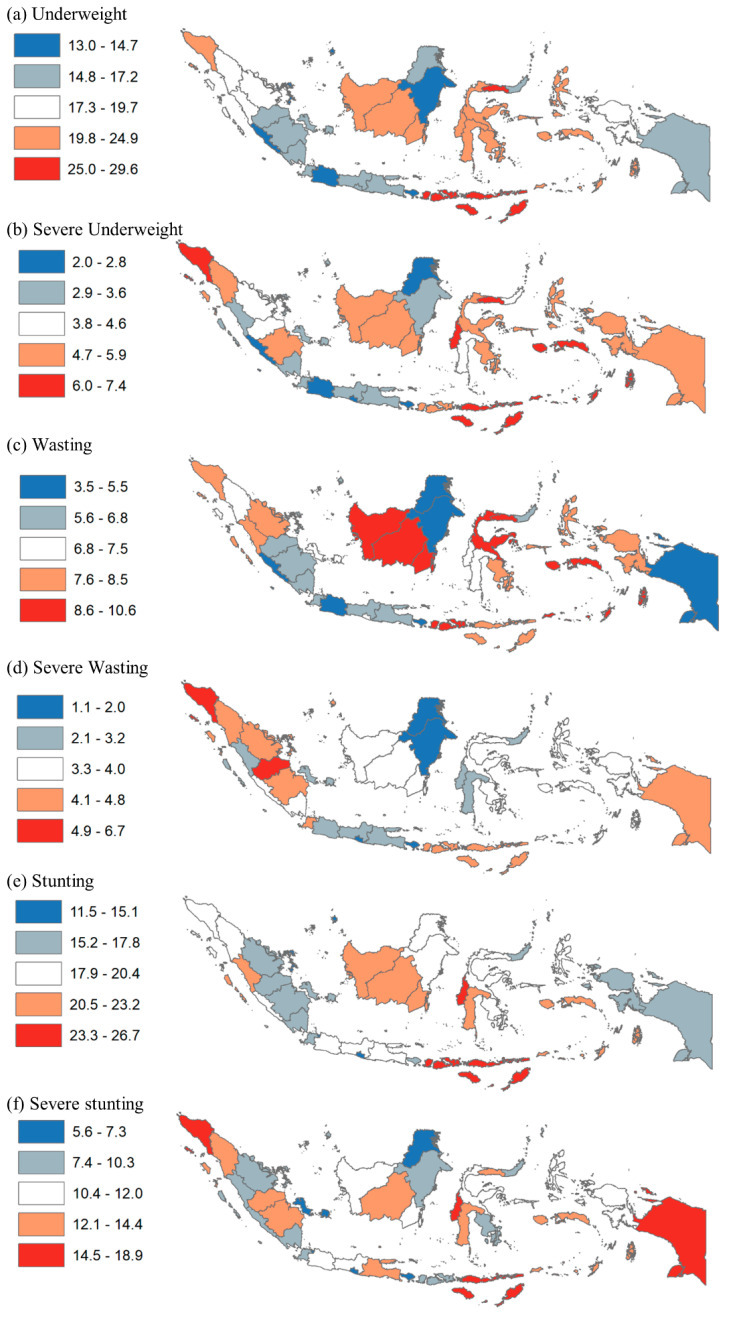
Disparity of Child Undernutrition by Province in Indonesia, 2018. Note: Values show prevalence of child undernutrition.

In terms of geographic disparity, Figure 2 shows the distribution of child undernutrition prevalence quintiles by province. The prevalence of underweight ranged from 13.0% to 29.6%; that of severe underweight ranged from 2.0% to 7.4%; that of wasting ranged from 3.5% to 10.6%; that of severe wasting ranged from 1.1% to 6.7%; that of stunting ranged from 11.5% to 26.7%; that of severe stunting ranged from 5.65 to 18.9%. For underweight and severe underweight, the prevalence was highest (quintile 5) in the Papua (including East Nusa Tenggara, West Nusa Tenggara, Maluku), northern Sulawesi (including Gorontalo), and northern Sumatera (including Aceh) regions. For wasting, the prevalence was highest in the northern Sulawesi (including Gorontalo), Kalimantan (including West Kalimantan), and Papua (including West Nusa Tenggara) regions. However, the prevalence was highest for severe wasting in the northern (including Aceh) and central Sumatera (including Jambi) regions. For stunting and severe stunting, the prevalence was highest in the Papua (including East Nusa Tenggara, West Nusa Tenggara, Papua), western Sulawesi (including West Sulawesi), and northern Sumatera (including Aceh) regions.

In terms of socioeconomic disparity, Table 1 shows the prevalence of child undernutrition by income at the provincial level. The top box shows provinces with the lowest poverty rates (i.e., wealthier) including Bali, South Kalimantan, Central Kalimantan, and Jakarta. The bottom box shows provinces with the higher poverty rates (i.e., poorer) including Papua, East Nusa Tenggara, Maluku, and Gorontalo. The prevalence with grey shades was higher than the national average for each indicator (column). Only two of the ten wealthiest provinces were consistently higher than average for all child undernutrition indicators. On the other hand, seven of ten poorest provinces were consistently higher than average for five or six child undernutrition indicators.

### 3.2. District Level

Table 2 shows the characteristics of districts and the prevalence of child undernutrition. There are 514 districts, consisting of 97 cities and 417 regencies. By region, most districts are in the Sumatera region (154 districts or 30.0% of total districts) and the Java region (128 districts or 24.9%). By income level, 79% of urban areas are relatively wealthy (in quintiles 4 and 5), while nearly half (47.2%) of rural areas are relatively poor (in quintiles 1 and 2). By the education level, 71.1% of urban areas have relatively high education (in quintiles 4 and 5), while nearly half (46.8%) of rural areas have relatively low education (in quintiles 1 and 2). In terms of child undernutrition (panel b), the prevalence of underweight and severe underweight is 19.1% and 4.6%; that of wasting and severe wasting is 7.2% and 3.7%; and that of for stunting and severe stunting is 19.7% and 12.3%, respectively. Moreover, the prevalence of child undernutrition is significantly higher in rural areas compared to urban areas. The prevalence of underweight and severe underweight in rural areas is higher by 1.21 (i.e., 19.8% divided by 16.3%) and 1.41 times, respectively, compared to urban areas. Similarly, the prevalence of wasting and severe wasting is higher in rural areas by 1.11 and 1.30 times, respectively; that of stunting and severe stunting is higher by 1.23 and 1.51 times, respectively, than in urban areas.

**Figure 3:**
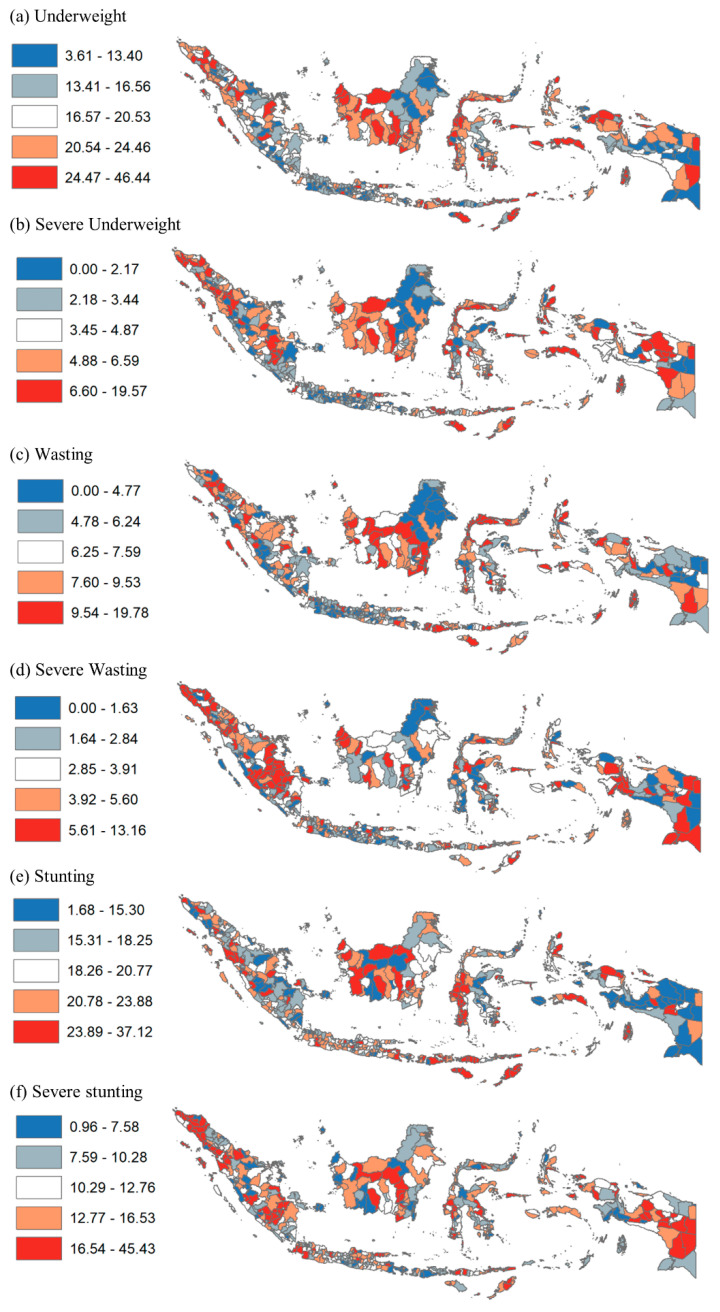
Disparity of Child Undernutrition by District in Indonesia, 2018. Note: Values show prevalence of child undernutrition.

In terms of geographic disparity, Figure 3 shows the disparity in child undernutrition at the district level by prevalence quintile. Results show more granularity by district than by province. For instance, many districts in Papua, Maluku, Central Sulawesi, West Kalimantan, Central Kalimantan, and Aceh provinces had the highest prevalence of underweight. Additionally, many districts in Papua, East Nusa Tenggara, West Kalimantan, Central Kalimantan, and South Sumatera provinces had the highest prevalence of severe wasting. Moreover, many districts in Papua, West Papua, Maluku, East Nusa Tenggara, West Sulawesi, West Kalimantan, Central Kalimantan, and Aceh provinces had the highest prevalence of stunting.

In terms of socioeconomic disparity, Table 3 and Table 4 provide the ten districts with the lowest and highest prevalence of child undernutrition, respectively. The prevalence of underweight ranged from 3.6% in Tabanan regency (Bali province) to 46.4% in Sabu Raijua (East Nusa Tenggara); that of severe underweight ranged from 0% in Salatiga city (Central Java), Yogyakarta city (Yogyakarta), Kediri city (East Java), Yahukimo (Papua), Tabanan (Bali), Pacitan (East Java), and Sumedang (West Java) to 19.6% in Aceh Selatan (Aceh). The prevalence of wasting ranged from 0% in Tolikara, Intan Jaya, Yahukimo (Papua), and Bener Meriah (Aceh) to 19.8% in Buton (Southeast Sulawesi); that of severe wasting ranged from 0% in 12 districts including Kepulauan Seribu (Jakarta), Malang city (East Java), and Aceh Tengah (Aceh) to 13.2% in Muara Enim (South Sumatera). Additionally, the prevalence of stunting ranged from 1.7% in Yahukimo (Papua) to 37.1% in Biak Nurfor (Papua); that of severe stunting ranged from 1.0% in Gianyar (Bali) to 45.4% in Dogiyai (Papua). By urban/rural, nearly all districts with the highest prevalence of child undernutrition are rural, but several districts with the lowest prevalence were urban. By income, the average poverty rates among the ten districts with the highest prevalence of child undernutrition were up to 26%, while the rates among the districts with the lowest prevalence were up to 22%.

**Figure 4:**
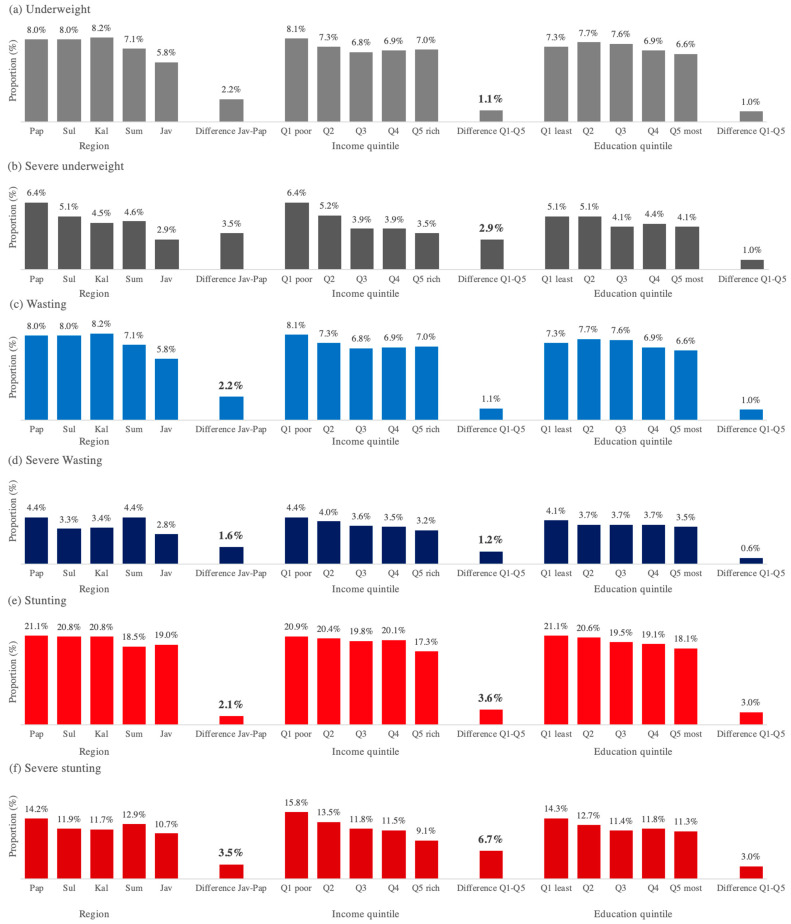
Note: Q = Quintile; Jav = Java region including Bali; Pap = Papua region including Maluku and Nusa Tenggara; Sul=Sulawesi region; Kal = Kalimantan region; Sum = Sumatera region. Income quintile used district-level poverty rate (Q1 = 20% of districts with highest poverty rate). Difference = Absolute difference between Papua and Java as well as Q1 and Q5. Appendix B shows the relative difference (ratio) between Papua and Java as well as Q1 and Q5. Appendix B also provides results stratified by urban/rural. Boldface difference values show statistical significance at a 5% level (see Appendix C for the regression outputs).

Figure 4 shows the associations between geographic and socioeconomic indicators (i.e., region, income, and education) and child undernutrition. The absolute (relative) values indicate the difference (ratio) between the Papua v Java regions and quintile 1 vs. quintile 5 for income and education level. We provide the absolute difference in Figure 4 and the relative difference in Appendix B. By region, districts in the Papua region had a significantly higher prevalence of underweight and wasting than those in the Java region. Districts in Papua had 44%, 121%, 38%, and 57% higher prevalence of underweight, severe underweight, wasting, and severe wasting; see Appendix B. Similarly, the poorest districts had a significantly higher prevalence of underweight, wasting, and stunting than the wealthiest districts. Poorest districts had 30%, 83%, 16%, 21%, and 74% higher prevalence of underweight, severe underweight, wasting, stunting, and severe stunting. These results were similar among rural districts (see Appendix B, Figure 4. Geographic and socioeconomic disparity in child undernutrition).

## 4. Discussion

Our study found a high prevalence of undernutrition among children under five years in Indonesia. The prevalence of underweight, wasting, and stunting were 19.1%, 7.2%, and 19.7%, respectively; also, severe underweight, severe wasting, and severe stunting were 4.6%, 3.7%, and 12.3%. Compared to the 2020 joint estimates by the World Bank/WHO/UNICEF, the prevalence of wasting in Indonesia was higher than the global average (6.7%) but lower than that among lower-middle-income countries (9.9%) in 2020. Similarly, the prevalence of severe wasting was higher than the global average (2.0%) and that among lower-middle-income countries (2.8%). However, the prevalence of stunting was slightly lower than the global average (22%) and that among lower-middle-income countries (29.1%) [23].

Our study also found a huge geographic and socioeconomic disparity in child undernutrition across districts in Indonesia. The prevalence of child undernutrition was significantly higher in rural areas (regents) than that in urban areas (cities) ranging from 1.11 times higher for wasting to 1.51 times higher for severe stunting. These findings align with previous studies in LMICs. A study on the disparity in child undernutrition in Cambodia found that the prevalence of underweight, wasting, and stunting was 1.59 times, 1.29 times, and 1.45 times higher in rural areas, respectively, compared to urban areas [17]. Studies in Nigeria and other countries in Sub-Saharan Africa also had similar findings [6,10].

Moreover, the prevalence of child undernutrition was significantly higher in Indonesia’s least developed region than in the most developed region. The prevalence of child nutrition in the Papua region (including Papua, Maluku, and Nusa Tenggara region) was up to 2.21 times (for underweight) higher than that in the Java and Bali region. This disparity is similar to the global trend. For instance, the prevalence of stunting among children under five was the highest in the least developed low-income countries (34.6%) and lowest in the most developed high-income countries (3.4%) in 2020 [4]. At the country level, a study on the disparity in child undernutrition across 640 districts in India found that the prevalence of stunting was higher among poorer populations in northern India compared to eastern and southern parts of India [7].

The significantly higher burden of child undernutrition in rural areas and Indonesia’s least developed region is also because those areas are relatively poorer and least educated at the district level. The prevalence of child undernutrition in the poorest districts was up to 1.83 times higher (for severe underweight) than that in the wealthiest districts. Even among rural districts (regencies) that had a lower income level than urban districts (cities), the prevalence of child undernutrition in the poorest rural districts was significantly higher. This aligns with previous studies in Bangladesh, Guatemala, and countries in sub-Saharan Africa [6,11,13,19].

For policy, our findings support the idea that governments in Indonesia and other countries with similar settings should put more effort into reducing child undernutrition in rural districts, districts in the least developed regions, and those in the poorest areas. While stunting reduction is currently among the top national priorities in Indonesia, the policies and interventions should not be “one size fits all”, given the huge geographic and socioeconomic disparity in child undernutrition in the country. A combination of top-down and bottom-up interventions is ideal for considering local situations, knowledge, and practices related to social determinants of child undernutrition such as safe water access, sanitation, and the quantity and quality of available food [24]. More efforts are needed to ensure improvement on each determinant. For instance, a study on access to improved sanitation in Indonesia found that households living in rural areas were 11.35% less likely to have access to improved sanitation facilities than those residing in urban areas [25]. Efforts through more community empowerment may also be helpful. A study comparing the household cash transfers (PKH) and community cash transfers (Generasi) programs for the poorest Indonesians found that both programs increased child food consumption, particularly of protein-rich items such as milk and fish, by up to 19% and 14% for PKH and Generasi, respectively. Additionally, PKH significantly reduced the probability of severe wasting by 41%, and Generasi significantly reduced the probability of being severely underweight by 47% [26].

Moreover, a “health in all policies” approach is needed to ensure effective efforts towards improving other social determinants of child undernutrition. They include programs to provide additional income (including conditional and unconditional cash transfers) and health insurance coverage (especially among the poor) [24,27]. More efforts are needed to ensure the improvement of those determinants as well. For instance, a study found that the impact of PKH cash transfer program on child basic vaccination was more prominent in urban areas compared to rural areas [28]. Additionally, a study on the disparity of health insurance ownership in Indonesia showed that those in urban areas were 2.32 times more likely to own health insurance than those in rural areas [29].

Our study has two limitations. First, data on child undernutrition by sex were not available for our analysis, which limited the disparity analysis among boys and girls. Second, our study used cross-sectional data on the prevalence of child undernutrition, which limited our analysis to assessing the temporal effect of association. Regardless of these limitations, our findings have important policy implications for Indonesia and other countries with similar settings.

## Figures and Tables

**Table 1:** Prevalence of child undernutrition by province in Indonesia, 2018.

		Prevalence					
	Poverty		Severe		Severe		Severe
	Rates	Underweight	Underweight	Wasting	Wasting	Stunting	Stunting
	(1)	(2)	(3)	(4)	(5)	(6)	(7)
Bali	4.5%	13.1%	2.0%	4.4%	1.9%	16.3%	5.6%
South Kalimantan	4.8%	24.5%	5.5%	9.2%	3.9%	21.1%	12.0%
Central Kalimantan	5.0%	21.8%	5.5%	9.9%	4.0%	21.3%	12.7%
Jakarta	5.0%	14.3%	2.3%	6.2%	3.9%	11.5%	6.2%
Banten	5.3%	16.2%	3.7%	5.9%	4.6%	17.0%	9.6%
Bangka Belitung	5.4%	17.0%	3.4%	7.1%	2.8%	16.1%	7.3%
West Sumatera	6.6%	18.9%	3.5%	8.4%	2.9%	21.7%	9.6%
North Kalimantan	7.0%	16.8%	2.4%	3.6%	1.1%	20.1%	6.8%
East Kalimantan	7.1%	14.7%	3.2%	5.5%	2.0%	19.0%	10.2%
Riau Islands	7.6%	13.0%	3.2%	6.7%	4.5%	15.1%	8.5%
Jambi	7.8%	15.7%	3.8%	6.3%	5.7%	16.8%	13.4%
North Maluku	7.9%	22.2%	5.6%	7.9%	3.9%	20.4%	11.0%
West Java	7.9%	13.2%	2.6%	5.2%	3.2%	19.4%	11.7%
West Kalimantan	8.1%	23.8%	5.2%	10.3%	4.0%	21.9%	11.4%
North Sulawesi	8.5%	15.4%	4.2%	6.7%	2.9%	15.7%	9.8%
Riau	8.8%	18.3%	4.3%	8.0%	4.2%	17.1%	10.3%
South Sulawesi	9.8%	22.9%	4.6%	7.5%	2.6%	23.2%	12.5%
West Sulawesi	10.3%	24.7%	6.3%	7.3%	3.2%	25.4%	16.2%
East Java	10.9%	16.8%	3.4%	6.3%	2.9%	19.9%	12.9%
Central Java	10.9%	16.8%	3.1%	5.8%	2.7%	20.1%	11.2%
North Sumatera	11.3%	19.7%	5.4%	7.5%	4.6%	19.2%	13.2%
Lampung	12.6%	15.9%	3.1%	6.8%	3.9%	17.7%	9.6%
Jogyakarta	12.7%	15.5%	2.5%	7.2%	1.2%	15.1%	6.3%
Southeast Sulawesi	13.0%	22.0%	5.6%	8.5%	3.4%	18.6%	10.1%
South Sumatera	13.1%	17.2%	4.9%	6.7%	4.7%	17.2%	14.4%
Central Sulawesi	14.6%	23.5%	4.8%	9.2%	3.7%	20.4%	11.9%
West Nusa Tenggara	14.8%	26.4%	5.9%	10.0%	4.4%	24.3%	9.2%
Bengkulu	15.0%	13.2%	2.8%	4.8%	3.5%	18.2%	9.8%
Aceh	16.4%	23.5%	6.7%	7.8%	6.7%	19.0%	18.9%
Gorontalo	16.8%	26.2%	6.8%	10.6%	3.8%	19.8%	12.7%
Maluku	21.8%	24.9%	7.4%	9.1%	4.1%	21.6%	12.5%
East Nusa Tenggara	22.0%	29.6%	7.3%	8.3%	4.6%	26.7%	16.0%
West Papua	26.5%	19.2%	5.2%	8.3%	3.9%	16.1%	11.7%
Papua	29.4%	16.6%	5.2%	5.5%	4.8%	17.8%	15.3%
AVERAGE		19.2%	4.4%	7.3%	3.6%	19.1%	11.2%

Note: Ordered by the average poverty rates (column 1), the provinces in the top box are richest and those in the bottom box are poorest. Shaded values show higher than the national average for each risk factor.

**Table 2:** Characteristics of districts and child undernutrition.

	All		Urban		Rural		Difference
	n	%	n	%	n	%	%
	(1)	(2)	(3)	(4)	(5)	(6)	(7) = (6 − 4)
(a) Characteristics (#)							
Sample size district	514	100%	97	100%	417	100%	0%
Region							
Papua	95	18.5%	9	9.3%	86	20.6%	11.3%
Java	128	24.9%	35	36.1%	93	22.3%	−13.8%
Sumatera	154	30.0%	33	34.0%	121	29.0%	−5.0%
Kalimantan	56	10.9%	9	9.3%	47	11.3%	2.0%
Sulawesi	81	15.8%	11	11.3%	70	16.8%	5.4%
	514		97		417		
Income/poverty							
Q1 poor	102	19.8%	3	3.1%	99	23.7%	20.6%
Q2	103	20.0%	5	5.2%	98	23.5%	18.3%
Q3	103	20.0%	13	13.4%	90	21.6%	8.2%
Q4	103	20.0%	22	22.7%	81	19.4%	−3.3%
Q5 rich	103	20.0%	54	55.7%	49	11.8%	−43.9%
	514		97		417		
Education							
Q1 least	103	20.0%	0	0.0%	103	24.7%	24.7%
Q2	103	20.0%	11	11.3%	92	22.1%	10.7%
Q3	103	20.0%	17	17.5%	86	20.6%	3.1%
Q4	103	20.0%	29	29.9%	74	17.7%	−12.2%
Q5 most	102	19.8%	40	41.2%	62	14.9%	−26.4%
	514		97		417		
(b) Child undernutrition (%)							
Underweight	n/a	19.1%	n/a	16.3%	n/a	19.8%	3.5% *
Severe underweight	n/a	4.6%	n/a	3.4%	n/a	4.8%	1.4% *
Wasting	n/a	7.2%	n/a	6.6%	n/a	7.3%	0.7% *
Severe wasting	n/a	3.7%	n/a	3.0%	n/a	3.9%	0.9% *
Stunting	n/a	19.7%	n/a	16.6%	n/a	20.4%	3.8% *
Severe stunting	n/a	12.3%	n/a	8.7%	n/a	13.1%	4.4% *

Note: Q = Quintile, n = number, % = proportion of column total, Urban = City, Rural = Regency. Data on district characteristics are from the World Bank, and data on CVD risk factors are from Indonesia Basic Health Survey 2018. Values with an asterisk (*) show statistical significance at 5% level (see Appendix A for the regression outputs).

**Table 3:** Ten districts with the LOWEST prevalence of child undernutrition in Indonesia.

	Prevalence	Province	Region	Urban	Poverty	Education	Pop (000)
	(1)	(2)	(3)	(4)	(5)	(6)	(7)
(a) Underweight							
Kab Tabanan	3.6%	Bali	Java	Rural	4%	81%	436
Kab Klungkung	5.2%	Bali	Java	Rural	6%	77%	176
Kota Tangerang Selatan	5.4%	Banten	Java	Urban	2%	73%	1539
Kota Tomohon	5.4%	North Sulawesi	Sulawesi	Urban	6%	71%	100
Kab. Puncak	5.9%	Papua	Papua	Rural	38%	9%	103
Kab. Sumedang	6.5%	West Java	Java	Rural	10%	43%	1137
Kota Bekasi	6.5%	West Java	Java	Urban	4%	71%	2709
Kab. Pegunungan Bintang	7.3%	Papua	Papua	Rural	31%	21%	72
Kab. Minahasa	7.4%	North Sulawesi	Sulawesi	Rural	7%	65%	329
Kota Bontang	7.4%	East Kalimantan	Kalimantan	Urban	5%	64%	166
AVERAGE					**11%**	**58%**	**677**
(b) Severe underweight							
Kota Salatiga	0%	Central Java	Java	Urban	5%	62%	184
Kota Yogyakarta	0%	Yogjakarta	Java	Urban	7%	73%	412
Kota Kediri	0%	East Java	Java	Urban	8%	79%	280
Kab. Yahukimo	0%	Papua	Papua	Rural	39%	12%	181
Kab Tabanan	0%	Bali	Java	Rural	4%	81%	436
Kab. Pacitan	0%	East Java	Java	Rural	14%	67%	551
Kab. Sumedang	0%	West Java	Java	Rural	10%	43%	1137
Kab. Tulung Agung	0.3%	East Java	Java	Rural	7%	63%	1021
Kota Depok	0.3%	West Java	Java	Urban	2%	75%	2100
kab. Semarang	0.3%	Central Java	Java	Rural	7%	56%	1000
AVERAGE					**10%**	**61%**	**730**
(c) Wasting							
Kab. Tolikara	0%	Papua	Papua	Rural	33%	34%	131
Kab. Intan Jaya	0%	Papua	Papua	Rural	43%	9%	46
Kab Bener Meriah	0%	Aceh	Sumatera	Rural	20%	67%	137
Kab. Yahukimo	0%	Papua	Papua	Rural	39%	12%	181
Kab. Kutai Barat	0.5%	East Kalimantan	Kalimantan	Rural	9%	60%	146
Kab. Malinau	0.7%	North Kalimantan	Kalimantan	Rural	8%	67%	77
Kab. Nduga	0.7%	Papua	Papua	Rural	38%	9%	94
Kab Klungkung	1.1%	Bali	Java	Rural	6%	77%	176
Kab Pesisir Barat	1.5%	Lampung	Sumatera	Rural	15%	72%	150
Kab Tabanan	1.7%	Bali	Java	Rural	4%	81%	436
AVERAGE					**22%**	**49%**	**157**
(d) Severe wasting							
Kep Seribu	0%	Jakarta	Java	Rural	12%	71%	23
Kota Malang	0%	East Java	Java	Urban	4%	65%	851
Kab Aceh Tengah	0%	Aceh	Sumatera	Rural	16%	73%	196
Kab. Purbalingga	0%	Central Java	Java	Rural	16%	55%	898
Kab. Morowali Utara	0%	Central Sulawesi	Sulawesi	Rural	16%	71%	117
Kota Tomohon	0%	North Sulawesi	Sulawesi	Urban	6%	71%	100
Kab. Boven Digul	0%	Papua	Papua	Rural	20%	35%	63
Kab. Intan Jaya	0%	Papua	Papua	Rural	43%	9%	46
Kota Samarinda	0%	East Kalimantan	Kalimantan	Urban	5%	66%	811
Kab. Jayawijaya	0%	Papua	Papua	Rural	39%	67%	206
AVERAGE					**18%**	**58%**	**331**
(e) Stunting							
Kab. Yahukimo	1.7%	Papua	Papua	Rural	39%	12%	181
Kab. Nagan Raya	3.9%	Aceh	Sumatera	Rural	19%	68%	155
Kab. Mambramo Tengah	7.0%	Papua	Papua	Rural	37%	54%	46
Kab. Sarolangun Bangko	7.9%	Jambi	Sumatera	Rural	9%	59%	278
Kab. Kolaka Timur	8.0%	Southeast Sulawesi	Sulawesi	Rural	14%	64%	178
Kab. Mambramo Raya	8.0%	Papua	Papua	Rural	30%	51%	21
Kota Sabang	8.9%	Aceh	Sumatera	Urban	16%	80%	33
Kab. Sintang	8.9%	West Kalimantan	Kalimantan	Rural	10%	45%	396
Kota Pangkal Pinang	9.1%	Bangka Belitung	Sumatera	Urban	5%	69%	196
Kab. Minahasa	9.2%	North Sulawesi	Sulawesi	Rural	7%	65%	329
AVERAGE					**19%**	**57%**	**181**
(f) Severe stunting							
Kab. Gianyar	1.0%	Bali	Java	Rural	4%	77%	495
Kab Belitung Timur	1.2%	Bangka Belitung	Sumatera	Rural	7%	62%	119
Kab. Buton Tengah	2.2%	Southeast Sulawesi	Sulawesi	Rural	15%	80%	89
Kab Tabanan	2.3%	Bali	Java	Rural	4%	81%	436
Kab. Natuna Kep	2.5%	Riau Islands	Sumatera	Rural	5%	70%	74
Kota Tangerang	3.1%	Banten	Java	Urban	5%	65%	2043
Kota Tangerang Selatan	3.1%	Banten	Java	Urban	2%	73%	1539
Kota Sawahlunto	3.3%	West Sumatera	Sumatera	Urban	2%	70%	60
Kota Metro	3.3%	Lampung	Sumatera	Urban	9%	83%	158
Kab. Minahasa Tenggara	3.4%	North Sulawesi	Sulawesi	Rural	13%	63%	104
AVERAGE					**7%**	**72%**	**512**

Note: Urban = City, Rural = Regency; Pop = Population. The districts are ordered by prevalence of child undernutrition (column 1). Boldface values show the average.

**Table 4:** Ten districts with the HIGHEST prevalence of child undernutrition in Indonesia, 2018.

	Prevalence	Province	Region	Urban	Poverty	Education	Pop (000)
(a) Underweight							
Sabu Raijua	46.4%	East Nusa Tenggara	Papua	Rural	31%	69%	86
Kab. Timor Tengah Selatan	42.8%	East Nusa Tenggara	Papua	Rural	28%	52%	459
Malaka	38.8%	East Nusa Tenggara	Papua	Rural	16%	58%	180
Kab. Gorontalo Utara	38.7%	Gorontalo	Sulawesi	Rural	19%	60%	111
Kab. Aceh Selatan	37.9%	Aceh	Sumatera	Rural	14%	72%	225
Rote Ndao	37.5%	East Nusa Tenggara	Papua	Rural	28%	51%	147
Sumba Tengah	36.1%	East Nusa Tenggara	Papua	Rural	35%	44%	68
Kab. Supiori	35.9%	Papua	Papua	Rural	39%	57%	18
Kab. Nias Barat	35.5%	North Sumatera	Sumatera	Rural	27%	80%	85
Kota Bima	34.2%	West Nusa Tenggara	Papua	Urban	9%	79%	159
AVERAGE					**25%**	**62%**	**154**
(b) Severe underweight							
Kab. Aceh Selatan	19.6%	Aceh	Sumatera	Rural	14%	72%	225
Sabu Raijua	17.1%	East Nusa Tenggara	Papua	Rural	31%	69%	86
Kab. Timor Tengah Selatan	16.4%	East Nusa Tenggara	Papua	Rural	28%	52%	459
Kab. Supiori	14.5%	Papua	Papua	Rural	39%	57%	18
Kab. Simeulue	14.2%	Aceh	Sumatera	Rural	20%	81%	89
Rote Ndao	12.6%	East Nusa Tenggara	Papua	Rural	28%	51%	147
Kab. Kep Yapen	12.6%	Papua	Papua	Rural	27%	55%	91
Kab. Nias Barat	12.6%	North Sumatera	Sumatera	Rural	27%	80%	85
Kab. Pulau Taliabu	12.3%	North Maluku	Papua	Rural	7%	58%	51
Kab. Waropen	12.2%	Papua	Papua	Rural	31%	61%	28
AVERAGE					**25%**	**63%**	**128**
(c) Wasting							
Kab. Buton	19.8%	Southeast Sulawesi	Sulawesi	Rural	14%	69%	98
Kota Palangka Raya	19.7%	Central Kalimantan	Kalimantan	Urban	3%	54%	259
Kab. Bone Bolango	19.6%	Gorontalo	Sulawesi	Rural	17%	61%	153
Kayong Utara	18.4%	West Kalimantan	Kalimantan	Rural	10%	56%	105
Kab. Teluk Wondama	18.0%	West Papua	Papua	Rural	33%	39%	30
Kab. Tabalong	15.3%	South Kalimantan	Kalimantan	Rural	6%	61%	239
Melawi	14.5%	West Kalimantan	Kalimantan	Rural	13%	41%	196
Kab. Sintang	14.4%	West Kalimantan	Kalimantan	Rural	10%	45%	396
Kab. Maluku Tenggara Barat	14.0%	Maluku	Papua	Rural	28%	50%	110
Sekadau	13.7%	West Kalimantan	Kalimantan	Rural	6%	45%	193
AVERAGE					**14%**	**52%**	**178**
(d) Severe wasting							
Kab. Muara Enim	13.2%	South Sumatera	Sumatera	Rural	13%	65%	600
Kab. Aceh Jaya	12.3%	Aceh	Sumatera	Rural	14%	74%	86
Kab. Merauke	12.2%	Papua	Papua	Rural	11%	65%	216
Kab. Buton Tengah	12.0%	Southeast Sulawesi	Sulawesi	Rural	15%	80%	89
Kab. Puncak Jaya	11.6%	Papua	Papua	Rural	36%	21%	115
Kab. Aceh Selatan	10.9%	Aceh	Sumatera	Rural	14%	72%	225
Kab. Tulang Bawang Barat	10.8%	Lampung	Sumatera	Rural	8%	52%	264
Kota Bima	10.4%	West Nusa Tenggara	Papua	Urban	9%	79%	159
Kab. Teluk Bintuni	10.4%	West Papua	Papua	Rural	31%	56%	59
Kab. Pidie	10.3%	Aceh	Sumatera	Rural	20%	74%	418
AVERAGE					**17%**	**64%**	**223**
(e) Stunting							
Kab. Biak Numfor	37.1%	Papua	Papua	Rural	26%	62%	139
Manggarai	32.6%	East Nusa Tenggara	Papua	Rural	21%	51%	319
Kab. Timor Tengah Utara	32.2%	East Nusa Tenggara	Papua	Rural	22%	54%	244
Kab. Nduga	32.2%	Papua	Papua	Rural	38%	9%	94
Kab. Kotawaringin Timur	32.2%	Central Kalimantan	Kalimantan	Rural	6%	46%	425
Kab. Kep Yapen	32.0%	Papua	Papua	Rural	27%	55%	91
Kab. Konawe Kepulauan	31.9%	Southeast Sulawesi	Sulawesi	Rural	17%	63%	32
Kab. Intan Jaya	31.8%	Papua	Papua	Rural	43%	9%	46
Kab. Sambas	31.8%	West Kalimantan	Kalimantan	Rural	9%	49%	523
Kab. Maybrat	31.6%	West Papua	Papua	Rural	33%	69%	37
AVERAGE					**24%**	**47%**	**195**
(f) Severe stunting							
Kab. Dogiyai	45.4%	Papua	Papmalnus	Rural	30%	39%	92
Kab. Nagan Raya	37.7%	Aceh	Sumatera	Rural	19%	68%	155
Kab. Nias	35.8%	North Sumatera	Sumatera	Rural	16%	62%	136
Kab. Paniayi	33.0%	Papua	Papmalnus	Rural	37%	25%	164
Kab. Yahukimo	32.6%	Papua	Papmalnus	Rural	39%	12%	181
Kab. Waropen	31.5%	Papua	Papmalnus	Rural	31%	61%	28
Kab. Lahat	28.2%	South Sumatera	Sumatera	Rural	16%	67%	393
Kab. Mambramo Tengah	27.7%	Papua	Papmalnus	Rural	37%	54%	46
Kab Bener Meriah	27.6%	Aceh	Sumatera	Rural	20%	67%	137
Kab. Aceh Timur	27.2%	Aceh	Sumatera	Rural	14%	58%	402
AVERAGE					**26%**	**51%**	**173**

Note: Urban = City, Rural = Regency; Pop = Population. The districts are ordered by child undernutrition prevalence (column 1). Boldface values show the average.

## Data Availability

The data presented in this study are available on request from the corresponding author. The data are not publicly available due to local policy.

## Appendix A

**Table A1:** Regression outputs for urban/rural differences.

	Underweight	Severe Underweight	Wasting	Severe Wasting	Stunting	Severe Stunting
VARIABLES	Coef	Coef	Coef	Coef	Coef	Coef
Rural	Reference					
Urban	−3.53 **	−1.42 **	−0.71 *	−0.89 **	−3.75 **	−4.41 **
Constant	19.79 **	4.82 **	7.33 **	3.90 **	20.40 **	13.15 **
Observations	513	513	513	513	513	513
R-squared	0.04	0.04	0.01	0.02	0.08	0.09

Note: Coef = OLS coefficients; Significance level ** *p* < 0.01, * *p* < 0.05.

## Appendix B

**Table A2:** Geographic and socioeconomic disparity in child undernutrition.

	All districts (n = 514)						Urban (n = 97)						Rural (n = 417)					
		Severe		Severe		Severe		Severe		Severe		Severe		Severe		Severe		Severe
	under Weight	under Weight	Wasting	Wasting	Stunting	Stunting	under Weight	under Weight	Wasting	Wasting	Stunting	Stunting	under Weight	under Weight	Wasting	Wasting	Stunting	Stunting
	(1)	(2)	(3)	(4)	(5)	(6)	(7)	(8)	(9)	(10)	(11)	(12)	(13)	(14)	(15)	(16)	(17)	(18)
Region																		
Papua	22.1%	6.4%	8.0%	4.4%	21.1%	14.2%	17.9%	4.1%	5.7%	3.7%	17.8%	7.7%	22.5%	6.7%	8.2%	4.5%	21.4%	14.9%
Sulawesi	22.1%	5.1%	8.0%	3.3%	20.8%	11.9%	20.1%	5.5%	7.2%	3.0%	18.5%	8.2%	22.4%	5.0%	8.2%	3.4%	21.1%	12.5%
Kalimantan	20.6%	4.5%	8.2%	3.4%	20.8%	11.7%	16.7%	3.7%	8.8%	1.9%	17.8%	8.7%	21.4%	4.6%	8.1%	3.7%	21.4%	12.3%
Sumatera	18.4%	4.6%	7.1%	4.4%	18.5%	12.9%	16.5%	3.3%	6.6%	3.5%	15.5%	9.5%	18.9%	4.9%	7.2%	4.6%	19.3%	13.9%
Java	15.4%	2.9%	5.8%	2.8%	19.0%	10.7%	14.4%	2.6%	6.2%	2.6%	16.5%	8.5%	15.8%	3.0%	5.6%	2.9%	19.9%	11.6%
Absolute	**6.7%**	**3.5%**	**2.2%**	**1.6%**	2.1%	3.5%	3.5%	1.5%	**−0.5%**	1.1%	1.3%	−0.8%	**6.7%**	**3.7%**	**2.6%**	**1.6%**	1.5%	3.3%
Relative	**1.44**	**2.21**	**1.38**	**1.57**	1.11	1.33	1.24	1.58	**0.92**	1.42	1.08	0.91	**1.42**	**2.23**	**1.46**	**1.55**	1.08	1.28
Income																		
Q1 poor	22.0%	6.4%	8.1%	4.4%	20.9%	15.8%	18.2%	5.4%	6.9%	2.3%	18.8%	11.0%	22.1%	6.4%	8.1%	4.5%	21.0%	15.9%
Q2	20.4%	5.2%	7.3%	4.0%	20.4%	13.5%	16.3%	4.4%	6.2%	2.9%	17.4%	8.2%	20.6%	5.2%	7.3%	4.0%	20.5%	13.7%
Q3	17.9%	3.9%	6.8%	3.6%	19.8%	11.8%	16.1%	3.1%	6.3%	3.5%	15.9%	9.9%	18.2%	4.0%	6.8%	3.6%	20.3%	12.1%
Q4	18.4%	3.9%	6.9%	3.5%	20.1%	11.5%	18.7%	3.7%	6.6%	3.3%	19.2%	9.7%	18.3%	3.9%	7.0%	3.6%	20.4%	11.9%
Q5 rich	16.9%	3.5%	7.0%	3.2%	17.3%	9.1%	15.2%	3.2%	6.7%	2.8%	15.6%	8.0%	18.8%	3.8%	7.2%	3.6%	19.1%	10.3%
Absolute	**5.1%**	**2.9%**	**1.1%**	1.2%	**3.6%**	**6.7%**	3.0%	2.2%	0.2%	−0.5%	3.2%	3.0%	**3.3%**	**2.6%**	0.9%	0.9%	**1.9%**	**5.6%**
Relative	**1.30**	**1.83**	**1.16**	1.38	**1.21**	**1.74**	1.20	1.69	1.03	0.82	1.21	1.38	**1.18**	**1.68**	1.13	1.25	**1.10**	**1.54**
Education																		
Q1 least	19.7%	5.1%	7.3%	4.1%	21.1%	14.3%	n/a	n/a	n/a	n/a	n/a	n/a	19.7%	5.1%	7.3%	4.1%	21.1%	14.3%
Q2	21.0%	5.1%	7.7%	3.7%	20.6%	12.7%	19.6%	4.4%	9.4%	3.4%	17.0%	8.1%	21.2%	5.1%	7.5%	3.8%	21.1%	13.3%
Q3	18.8%	4.1%	7.6%	3.7%	19.5%	11.4%	15.2%	2.6%	6.9%	2.5%	16.5%	7.9%	19.5%	4.4%	7.7%	3.9%	20.1%	12.1%
Q4	18.6%	4.4%	6.9%	3.7%	19.1%	11.8%	15.5%	3.6%	5.9%	3.3%	16.7%	8.9%	19.8%	4.8%	7.3%	3.8%	20.1%	13.0%
Q5 most	17.4%	4.1%	6.6%	3.5%	18.1%	11.3%	16.3%	3.3%	6.3%	2.9%	16.6%	9.1%	18.2%	4.6%	6.8%	3.9%	19.0%	12.7%
Absolute	2.3%	1.0%	0.7%	0.6%	3.0%	3.0%	3.3%	1.1%	**3.1%**	0.5%	0.4%	−1.0%	1.5%	0.5%	0.5%	0.2%	2.1%	1.6%
Relative	1.13	1.24	1.11	1.17	1.17	1.27	1.20	1.33	**1.49**	1.17	1.02	0.89	1.08	1.11	1.07	1.05	1.11	1.13

Note: Q = Quintile; Java region includes Bali; Papua region includes Maluku and Nusa Tenggara. Income quintile used district-level poverty rate (Q1 = 20% of districts with highest poverty rate). Absolute (Relative) = Difference (Ratio) between Papua and Java as well as Q1 and Q5. For education, absolute (relative) was between Q1 and Q% except among urban (Q2 and Q5). Boldface values show statistically significance at 5% level (see Appendix C for the regression outputs).

## Appendix C

**Table A3:** Regression outputs for geographic and socioeconomic disparity in child undernutrition.

	Underweight	Severe Underweight	Wasting	Severe Wasting	Stunting	Severe Stunting
	Coef	Coef	Coef	Coef	Coef	Coef
Papua	Reference					
Java	−4.80 **	−2.51 **	−1.58 **	−1.20 **	−0.96	−0.23
Sumatera	−2.28 *	−1.02 **	−0.38	0.30	−1.74 *	1.38
Kalimantan	1.21	−0.52	0.98	−0.72	1.20	1.45
Sulawesi	0.91	−0.67	0.44	−0.88 *	0.07	−0.24
Rural	Reference					
Urban	−1.31	−0.39	−0.09	−0.57	−2.19 **	−2.17 **
Income						
Quintile 1 poor	Reference					
Quintile 2	−1.62	−0.77 *	−0.96 *	−0.10	−0.19	−2.20 **
Quintile 3	−2.75 **	−1.52 **	−1.07 *	−0.31	−0.47	−3.76 **
Quintile 4	−2.69 **	−1.74 **	−1.07 *	−0.37	−0.01	−4.12 **
Quintile 5 rich	−4.11 **	−2.04 **	−1.15 *	−0.33	−2.53 **	−5.81 **
Education						
Quintile 1 least	Reference					
Quintile 2	2.72 **	0.69	0.83	−0.16	0.38	−0.09
Quintile 3	0.39	−0.43	0.65	−0.28	−0.60	−1.56 *
Quintile 4	0.63	0.08	0.02	−0.26	−0.47	−0.98
Quintile 5 most	0.83	0.21	0.16	−0.43	−0.74	−0.90
Constant	22.31 **	6.84 **	8.06 **	4.72 **	21.65 **	16.14 **
Observations	513	513	513	513	513	513
R-squared	0.22	0.25	0.11	0.10	0.14	0.20

Note: Coef = OLS coefficients; Significance level ** *p* < 0.01, * *p* < 0.05.
